# Supplementary material for: Usability of myfood24 Healthcare and Mathematical Diet Optimisation in Clinical Populations: A Pilot Feasibility Randomised Controlled Trial
Source: Nutrients. 2022 Apr 23;14(9):1768. doi: 10.3390/nu14091768 (PMC9101756; doi:10.3390/nu14091768)
Supplement: Supplementary file 1 [file nutrients-14-01768-s001.zip › nutrients-1639299-supplementary.pdf]

nutrients

Supplementary Materials:

Usability of myfood24 Healthcare and Mathematical Diet Optimisation in Clinical Populations: A Pilot Feasibility Randomised Controlled Trial

Diane E. Threapleton [1], Sarah L. Beer [2], Dustin J. Foley [1,3], Lauren E. Gibson [2], Sarah Trevillion [4], Dermot Burke [5], Pete Wheatstone [1], Jacqui Gath [1], Nick Hex [6], Jo Setters [6], Darren C. Greenwood [3,7] and Janet E. Cade [1,2,*]

Table S1. Example dietary change suggestions.

Example number	Food description	Suggested change	Recorded intake	Suggested intake	Standard portion
1	Potato chips, microwaved	Add food	0g	153g	165g
1	Tea, white	Remove food	520ml	0ml	170ml
1	Potato chips, fried, takeaway	Remove food	153g	0g	210g
1	Ice cream, vanilla	Remove food	98.8ml	0ml	30ml
2	Eggs, chicken, scrambled	Eat less	113.5g	56.8g	25g
3	Cola drink	Eat less	500ml	250ml	330ml
3	Supermarket brand Beef Chilli	Eat less	300g	150g	300g
3	Chocolate bar	Remove food	55g	0g	64.5g
3	Bolognese sauce with meat, homemade	Eat less	116g	58g	240g
3	Luxury Hot Cross Buns	Remove food	156g	0g	312g
3	Yogurt, Greek style, plain	Remove food	150g	0g	200g
3	Unsalted Butter	Remove food	10g	0g	10g
4	Mayonnaise	Eat less	21g	10.5g	28g
4	All Butter Croissants	Remove food	71g	0g	71g
4	70% Dark Chocolate	Remove food	60g	0g	100g
5	Supermarket brand Beef Lasagne	Eat more	350g	700g	350g
5	Nutty Muesli	Eat more	50g	75g	50g
5	Caffe Mocha Coconut	Remove food	455ml	0ml	227ml
5	Vanilla Flavour Yogurt Drink	Remove food	250ml	0ml	375ml
5	Chocolate bar	Remove food	40g	0g	40g
5	Butter, salted	Remove food	12g	0g	250g
6	Cabernet Sauvignon	Eat less	500ml	250ml	125ml
6	Bacon And Egg Roll	Eat more	194g	388g	194g
6	Pie, Cottage, homemade	Eat more	236.2g	354.2g	310g
6	Fruit cordial	Remove food	330ml	0ml	375ml
6	Pain Aux Raisins	Remove food	120g	0g	120g
6	Chocolate bar	Remove food	40g	0g	40g
6	Lemon Curd	Remove food	15g	0g	15g
7	Bread, white, sliced	Eat more	98g	196g	31g
7	Eggs, chicken, whole, fried in sunflower oil	Eat more	180g	270g	60g
7	Pears, raw, flesh and skin	Eat more	160g	240g	160g
7	Stuffed Crust Meat Feast Pizza	Remove food	237g	0g	475g
7	Butter, spreadable 75-80% fat	Remove food	20g	0g	250g

Note, brand information has been removed.
